# Pyridoxal 5’-phosphate synthesis and salvage in Bacteria and Archaea: predicting pathway variant distributions and holes

**DOI:** 10.1099/mgen.0.000926

**Published:** 2023-02-02

**Authors:** Rémi Denise, Jill Babor, John A. Gerlt, Valérie de Crécy-Lagard

**Affiliations:** ^1^​ Department of Microbiology and Cell Sciences, Gainesville, USA; ^2^​ Department of Chemistry, Gainesville, USA; ^3^​ Genetics Institute, University of Florida, Gainesville, FL 32611, USA; ^†^​ Present address: APC Microbiome Ireland, University College Cork, Cork, Ireland

**Keywords:** B6 vitamers, Hidden Markov models, pyridoxal, pyridoxine, sequence similarity networks

## Abstract

Pyridoxal 5’-phosphate or PLP is a cofactor derived from B_6_ vitamers and essential for growth in all known organisms. PLP synthesis and salvage pathways are well characterized in a few model species even though key components, such as the vitamin B_6_ transporters, are still to be identified in many organisms including the model bacteria *Escherichia coli* or *Bacillus subtilis *. Using a comparative genomic approach, PLP synthesis and salvage pathways were predicted in 5840 bacterial and archaeal species with complete genomes. The distribution of the two known *de novo* biosynthesis pathways and previously identified cases of non-orthologous displacements were surveyed in the process. This analysis revealed that several PLP *de novo* pathway genes remain to be identified in many organisms, either because sequence similarity alone cannot be used to discriminate among several homologous candidates or due to non-orthologous displacements. Candidates for some of these pathway holes were identified using published TnSeq data, but many remain. We find that ~10 % of the analysed organisms rely on salvage but further analyses will be required to identify potential transporters. This work is a starting point to model the exchanges of B_6_ vitamers in communities, predict the sensitivity of a given organism to drugs targeting PLP synthesis enzymes, and identify numerous gaps in knowledge that will need to be tackled in the years to come.

## Data Summary

***Supplemental Material*** Methods, Analyses, and Figures (S1 to S6).

***Supplemental Data*** deposited in the Microbiology Society’s Figshare repository 10.6084/m9.figshare.20304351 [1].

**Impact Statement**

This analysis of the phylogenetic distribution of PLP synthesis and salvage genes is a massive update from the previously published data (from 690 species to 5840 species). It has changed our vision of the distribution of the bacterial DXP-dependent and DXP-independent pathways. In addition, we have identified key signature genes that can be used to predict the presence/absence of *de novo* and salvage pathways.

This work has identified several cases of non-orthologous replacement but also shows that many more still need to be identified. As a cautionary, not all families were analysed with the same depth because the memberships of large superfamily could not allow correct propagation without further experimental evidence. This work emphasizes that many genes still need to be identified even in the *de novo* pathways and is a starting point to guide further studies and potentially predict the range of potential antibacterial agents targeting PLP synthesis enzymes.

## Introduction

**Fig. 1.**
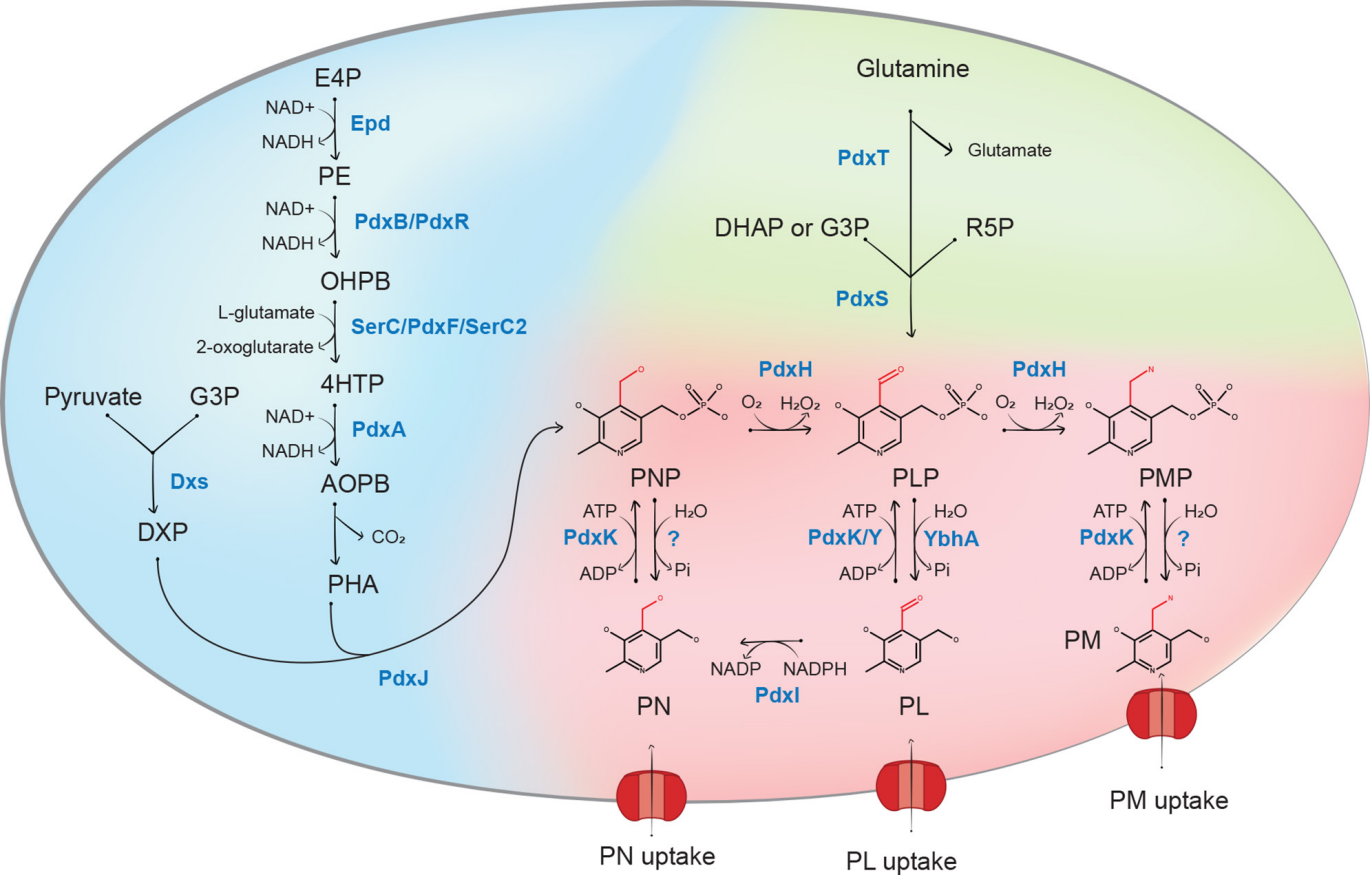
The vitamins are pyridoxal (PL), pyridoxal 5′-phosphate (PLP), pyridoxine (PN), pyridoxine 5′-phosphate (PNP), pyridoxamine (PM), pyridoxamine 5′-phosphate (PMP). The enzymes belonging to the DXP-dependent route are Epd, erythrose 4-phosphate dehydrogenase; PdxB or PdxR, 4-phosphoerythronate dehydrogenase; SerC (PdxF) or SerC2, 3-phosphoserine aminotransferase; PdxA, 4-phosphohydroxy-l-threonine dehydrogenase; PdxJ, PNP synthase; Dxs, 1-deoxyxylulose 5-phosphate synthase; PdxH, PNP oxidase. The enzymes in the DXP-dependent route are PdxS (PLP synthase subunit) and PdxT (glutaminase subunit). Finally, the salvage pathway is composed of PdxI, PL reductase; YbhA, PLP phosphatase; PdxK, PL/ PM/PN kinase present in B. subtilis (also called ThiD2) and E. coli; PdxY, PL kinase. Abbreviation: E4P, erythrose 4-phosphate; PE, 4-phosphoerythronate; OHPB, 2-oxo-3-hydroxy-4-phosphobutanoate; 4HTP, 4-phospho-hydroxy-threonine; AOPB, 2-a mino-3-oxo-4-(phosphohydroxyl)-butyrate; PHA, 3-phosphohydroxy-1-aminoacetone; DXP, deoxyxylulose-5-phosphate; G3P, glyceraldehyde-3-phosphate; DHAP, dihydroxyacetone phosphate; R5P, Ribose-5-phosphate. Red radical in the chemical representation of the vitamers represents the difference between each vitamer. This figure is an adaptation of the figure from Richts *et al*. [7]. All EC numbers and protein family identifiers (Pfam or COG) are given in Table S1.

During the decade spanning 1935 to 1945, the six interconvertible vitamin B_6_ species (pyridoxal or PL, pyridoxine or PN, pyridoxamine or PM, and their 5’-phosphate forms) (Fig. 1) were characterized, and the central role of the pyridoxal 5’-phosphate (PLP) cofactor was established [2, 3]. Over 80 years later, the field remains vibrant with the recent discovery of novel diseases linked to PLP homeostasis [4, 5]. Yet, surprisingly for such a classical metabolic area, many fundamental questions remain to be solved. For example, the identities of B_6_ vitamer transporters are still mysterious in most species even the model organisms such as *Escherichia coli* and *Bacillus subtilis* [6–8]. In addition, how free levels of the reactive, hence toxic, PLP molecule are kept low in the cell while still being delivered in sufficient amounts to target enzymes remains to be determined [6, 7, 9].

More than 180 enzymes utilizing PLP as a cofactor are found across all organisms [10, 11], and around 1.5 % of bacterial and archaeal genes encode PLP-binding proteins [11]. These enzymes catalyse diverse reactions in many areas of metabolism, including transamination, decarboxylation, racemization, and beta- and gamma-elimination [9]. Furthermore, all sequenced bacteria to date harbour PLP-dependent enzymes, and even the synthetic minimal organism *Mycoplasma* JCVI-Syn3 encodes an essential PLP-dependent cysteine desulfurase (IscS) [12].

In contrast with the universality of PLP-dependent reactions, the PLP synthesis pathways vary along the bacterial tree. Some intracellular bacteria, such as *Mycoplasma capricolum,* lack known PLP synthesis or salvage genes and must directly transport the final phosphorylated form of the cofactor (PLP) [13]. Other bacteria are, like humans, auxotrophic for B_6_ vitamers and require two salvage enzymes (Fig. 1, Table S1, available in the online Supplementary Material), PNP/PMP oxidase (PdxH) and PL/PN/PM kinase. PL/PN/PM kinases can belong to the PdxK or PdxY subgroups of the COG2240 family [13] or to the ThiD2 (PdxK) subgroup of the COG0351 family [14], which vary mainly by their substrate-specificities.

However, most bacteria can like yeast and plants, salvage and synthesize PLP *de novo* [15, 16] (Fig. 1, Table S1). Two different biosynthesis pathways have been characterized in the last 30 years [15, 17, 18]. In the deoxyxylulose 5-phosphate (DXP)-dependent pathway, first described in the model Gram-negative *Escherichia coli* K12, DXP and 3-phosphohydroxy-1-aminoacetone (PHA) are condensed by pyridoxine 5’-phosphate synthase (PdxJ) to create PNP, which will be then oxidized to PLP by pyridoxine/pyridoxamine 5'-phosphate oxidase (PdxH) [9]. Hence in organisms with the DXP-dependent (DXP-D) pathway, PdxH is both a *de novo* synthesis and a salvage enzyme. PHA is synthesized in four steps from erythrose 4-phosphate (E4P). In *E. coli *, the enzymes involved are d-erythrose-4-phosphate dehydrogenase (Epd), erythronate-4-phosphate dehydrogenase (PdxB), the bifunctional phosphoserine/3-hydroxy-4-phospho-hydroxy-α-ketobutyrate aminotransferase (SerC/PdxF) that modifies both phosphoserine and 3-hydroxy-4-phospho-hydroxy-α-ketobutyrate, and 4-hydroxythreonine-4-phosphate dehydrogenase (PdxA). Two cases of non-orthologous displacements have already been reported for the DXP-D pathway enzymes. Phosphoserine transaminase activity is encoded in the cyanobacterium *Synechocystis* sp. PCC 6803 by the *sll1559* gene (SerC2) and is not a member of the SerC protein family (Table S1) [19]. It is likely that SerC2 is also involved in B_6_ synthesis, even if it is yet to be experimentally validated. The PdxB enzyme is replaced in some organisms, such as *Sinorhizobium meliloti,* by a non-orthologous enzyme PdxR (Uniprot Id F7XD40) [20]. This last enzyme should not be confused with the transcriptional regulator of the same name but of a different family that regulates PLP synthesis genes in several Gram-positive bacteria [21–25]. In the DXP-independent (DXP-I) pathway, the PLP synthase subunit (PdxS) and the glutaminase subunit (PdxT) of the PLP synthase complex use three precursors; d-ribose 5-phosphate (R5P), glutamine, and glyceraldehyde 3-phosphate (G3P) to make PLP directly (Fig. 1, Table S1) [6]. This pathway is found in the model Gram-positive *B. subtilis* 168 [7].

A few additional salvage enzymes have been identified. The *pdxI* gene encodes for an NADPH-dependent PL reductase, which converts PL into PN [26]. This enzyme, also annotated as pyridoxine 4′-dehydrogenase, belongs to the aldo-keto reductase family (COG0667). The *ybhA* gene encodes a PLP phosphatase belonging to the superfamily of haloacid dehalogenase (HAD)-like hydrolases (COG0561) [4, 5, 27–29]. Only three transporters of exogenous B_6_ vitamers or PLP have been characterized in any prokaryotic organism: PdxU (COG4720, PF07155), PdxU2/HmpT (COG4720, PF07155), and P5PA (COG1840, PF13531) [28–30]. However, substrates have only been verified for P5PA and PdxU2/HmpT, emphasizing that this crucial area of vitamin B_6_ metabolism is poorly studied.

Because of the conserved nature of vitamins, comparative genomic approaches that analyse the distribution of the known synthesis and salvage genes have turned out to be powerful tools [31–33]. These approaches have identified pathway holes, predicted non-orthologous replacements or alternate pathways in specific species, and evaluated potential antibacterial target candidates [34, 35]. In addition, these analyses can help identify and disambiguate paralogs [36, 37] or identify missing regulatory components or transporters [38]. However, the previous comparative genomic study focusing specifically on PLP synthesis in Bacteria is nearly 20 years old [39]. More recent studies focused only on model organisms such as *E. coli* [40] or *B. subtilis* [7], on model human gut bacteria [41], or on explicitly analysing PLP-dependent transcription factor regulons [28]. Therefore, it seemed timely to perform a systematic analysis of PLP synthesis and salvage in sequenced bacteria to address this knowledge gap across the current diversity of bacterial species.

Additionally, *de novo* PLP synthesis proteins are critical for the virulence of pathogenic bacteria [22, 42] and are therefore proposed as potential antibacterial targets as they are absent in the human host [43]. Analysing these genes' distribution in the major groups of pathogenic bacteria can help rank these targets and predict the host range of compounds targeting specific B_6_ synthesis enzymes. Finally, vitamin exchanges are turning out to be ecological drivers, and these can be modelled only if the corresponding pathways are correctly and exhaustively annotated in all species involved [3, 44, 45]. The predictions for PLP synthesis and salvage presented here can be used as foundations for modelling and experimental validations.

## Methods

### Data

We analysed 21 114 complete bacterial and archaeal genomes from NCBI RefSeq (ftp://ftp.ncbi.nlm.nih.gov/genomes/refseq/, last accessed in March 2021) [46], representing 5840 species of Bacteria and 288 species of Archaea. HMM profiles were extracted from the Panther database (version 15) [47]. TnSeq data was accessed on the Fitness browser https://fit.genomics.lbl.gov/cgi-bin/myFrontPage.cgi [48].

### General bioinformatic resources

For literature and sequence retrievals, the resources at NCBI (https://www.ncbi.nlm.nih.gov/) [49], UniProt (https://www.uniprot.org) [50], and BV-BRC (https://www.bv-brc.org/) [51] were routinely used. PaperBlast was used to find published papers on members of a given family (papers.genomics.lbl.gov/) [52]. Sequence Similarity Networks (SSNs) were generated with the Enzyme Function Initiative (EFI) suite of webtools (https://efi.igb.illinois.edu/) [53]. SSNs were visualized using Cytoscape [54]. Species phylogenetic trees were constructed using phyloT_v2 (https://phylot.biobyte.de/). Both species and protein phylogenetic trees were visualized with iTOL v6 [55] and annotated using KofAmKOALA v2021-04-01 [56]. Gene neighbourhood diagrams were generated using Gene Graphics [57].

### Detection of pathways

The genes of the PLP synthesis and salvage pathways were detected using hmmsearch (default parameters) from HMMer v3.3 (Nov 2019) [58]. This programme uses HMM profiles to identify proteins in each proteome. The supplemental methods describe in detail the generation of the HMM profiles used.

### Phylogenetic inference

Phylogenetic analyses based on protein sequences involved an inference of a guide tree using the GraphSplitting method [59] and an initial alignment of the sequences using MAFFT v7.453 [60] (‘linsi’ algorithm, using the tree of the gene as guide tree, option --treein). Multiple alignments were analysed using Noisy v1.5.12 (default parameters) [61] to select the informative sites. Maximum likelihood trees were inferred from the curated alignments using IQ-TREE v 2.1.2 [62] (options -allnni, -nstop 1000, -nm 100000). Node supports were evaluated using the options -bb 1000 for ultra-fast bootstraps and -alrt 1000 for SH-aLRT [63]. Finally, the best evolutionary models were selected with ModelFinder (option -MF, BIC criterion) [45].

### Finding homologs of PLP-dependent signature proteins

Blastp v2.12.0 [64, 65] was used to search for homologs of the IscS protein of *E. coli* (YP_026169.1) and the SufS of *Haloferax volcanii* (WP_049914909.1) in the genomes lacking PLP pathways (*de novo* or salvage). Then, in a second step, we clustered the proteins based on a specific threshold (e-value=0.01, a percentage of identity of 30%, and a coverage of 80%) using silix v1.3.0 [66]. All the proteins above this threshold were annotated as members of the IscS/SufS family. Blastp was also used to find homologs of the P5PA transporter protein in our genomes of interest, using four iterations of Psi-Blast with P5PA from *Actinobacillus pleuropneumoniae* (WP_005596767.1) as input query.

## Results and discussion

### Identification of predicted PLP synthesis and salvage genes

Analysis of the literature summarized in the introduction led to the capture of all experimentally validated PLP synthesis and salvage proteins (Table S1). Of note, the few known regulators, such as PdxR and PdxR2 a [21–25], were not covered in this study as they have been already analysed by the Rodionov Laboratory [28]. We set out to identify the orthologs of these proteins in 21 114 complete bacterial and archaeal genomes covering 5840 species available in RefSeq, a comprehensive, integrated, and non-redundant database [46] (Table S2). Using only complete genomes was critical to ensure that if a given orthologous family had no representative in a specific genome, it was not because it was only partially sequenced.

Identifying the PLP synthesis and salvage pathways in Bacteria and Archaea’s complete genomes is difficult as some of the pathway’s proteins belong to superfamilies. In these cases, the standard sequence similarity tools will detect several candidates in each genome. For example, PdxB is part of a large superfamily of dehydrogenases (phosphoglycerate dehydrogenase [67]), that can be present in up to 20 members per genome (Fig. S1). Another example is the PdxK and PdxY kinases of the salvage pathway. PdxK has been shown to phosphorylate all B_6_ vitamers while PdxY only phosphorylates pyridoxal (PL), but both are members of the same COG (COG2240) [68] and part of the Ribokinase superfamily (PF00294). Hence, these two proteins must be differentiated between themselves and other superfamily members.

Hidden Markov model (HMM) profiles are sensitive tools for remote protein similarity detection [48]. It was, therefore, better suited than BLASTp to identify proteins in phylogenetically distant organisms. However, identifying proteins in superfamilies using HMM profiles can lead to overpredictions. To separate and annotate all superfamily members in a given genome and minimize this problem, specific HMM profiles were used for every superfamily subgroup. HMM profiles for all members of the PLP synthesis and salvage proteins superfamilies were available in the PANTHER database (Table S3). This first set of HMM profiles was used to scan our set of RefSeq genomes as described in the Methods section. Ideally, we expected that for any given PLP synthesis protein, most genomes would only harbour one copy. Unfortunately, the results were very variable. For example, the number of PdxB and PdxJ proteins detected per genome varied from a median of three for PdxB, to a median of one for PdxJ (Fig. S1). The HMM profiles available in PANTHER were not stringent enough for our purpose and needed to be refined to be more specific.

**Fig. 2.**
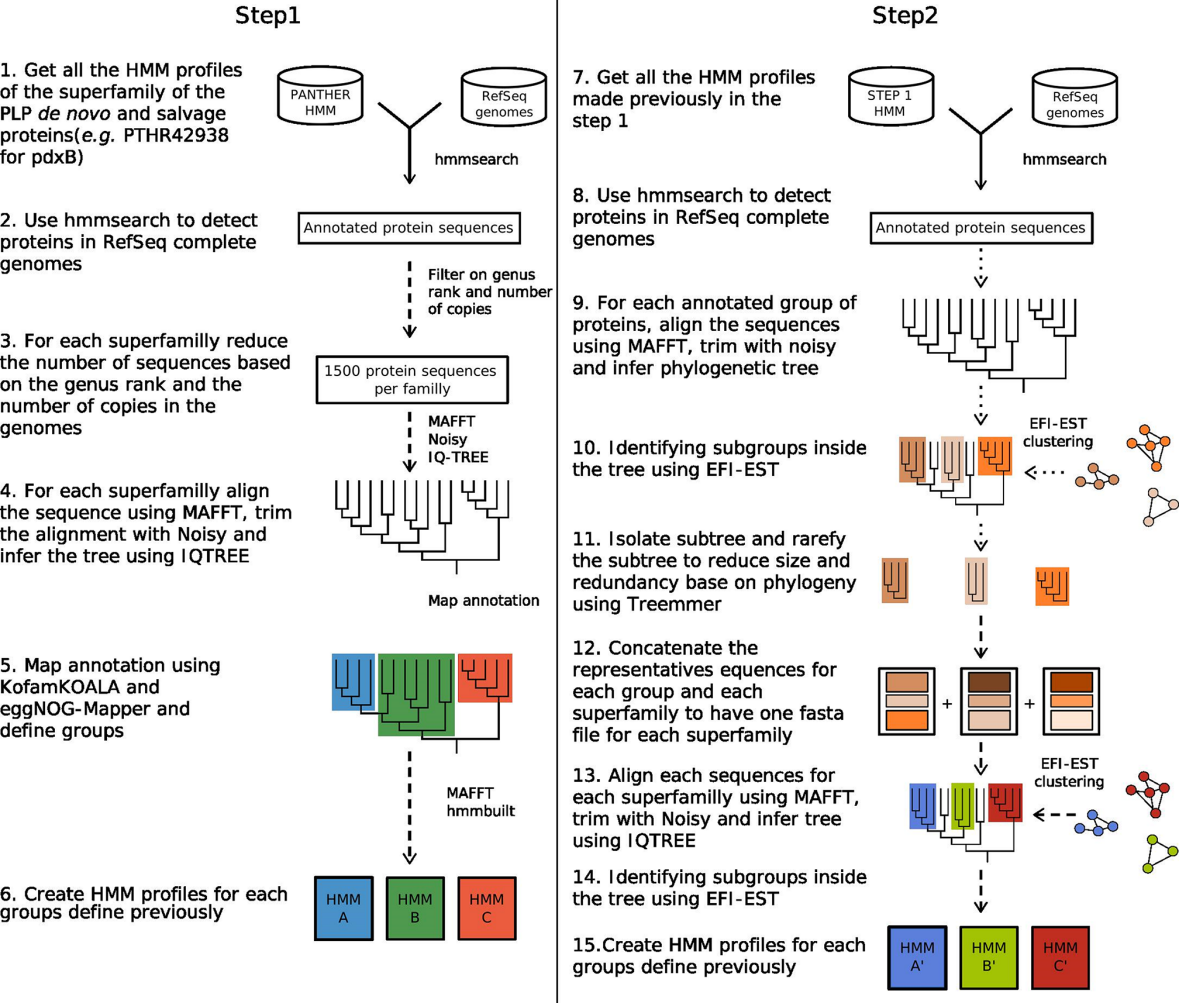
Summary of the process to create the HMM profiles used for the protein family detections. The straight arrows show that this step was made on the whole dataset. The dashed arrows indicate steps done for each superfamily. And dotted arrows represent steps done for each subgroup in the superfamily.

**Fig. 3.**
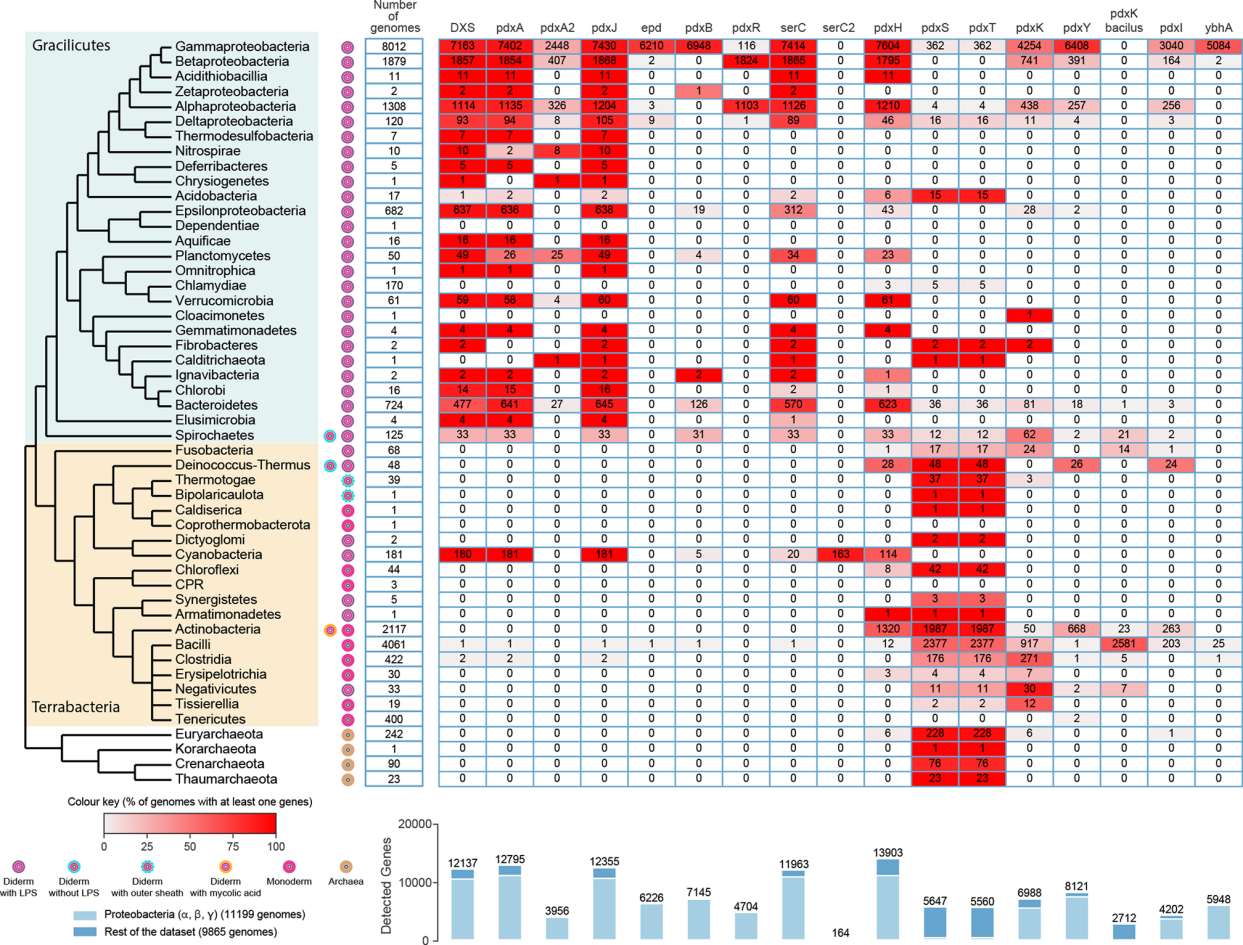
Taxonomic distribution of the PLP pathways' genes in Bacteria and Archaea. Cells indicate the number of genomes with at least one detected gene. The cell’s colour gradient represents the proportion of genomes with at least one gene in the clade. The bar plot shows the total number of detected genes. The bars are separated into alpha-, beta-, and gamma-proteobacteria versus the other clades. The cladogram symbolizes approximated relationships between the bacterial and archaeal taxa analysed in this study and was drawn using the phylogenetic analysis performed by Taib *et al.* [89].

A step-by-step refinement analysis, summarized in Fig. 2 and described in detail in the supplemental method section was then performed to generate HMM profiles stringent enough to distinguish the proteins involved in PLP biosynthesis from the other proteins in their respective superfamilies. This analysis led to the second set of 447 HMM protein profiles (listed in Table S4 and given in Sup. Data 2) used to query the distribution of PLP proteins encoded in complete prokaryotic genomes. The full analysis results are shown in Table S5 and summarized in Fig. 3. This strategy allowed us to differentiate close paralog cases such as the PdxA2 family involved in organic acid’s catabolism and not in PLP synthesis [36, 37] that are very difficult to annotate (Fig. S2) or the kinases that are part of large ribokinase superfamily (Fig. S3).

### Phylogenetic distribution of the de novo synthesis and salvage pathways in bacteria and archaea

Previous analyses performed with around 110 genomes [39] had reported that the DXP-D pathway was mainly present in gamma-proteobacteria, and the distribution of the DXP-I pathway was not analysed. To revise this analysis with more genomes, we identified signature genes for each pathway. We defined a set of criteria to call for the presence of these pathways in each genome. Indeed, not all genes are equivalent when predicting pathways in genomes. Some genes can be shared with other pathways (e.g., *serC* is used in PLP and serine synthesis). Members of non-orthologous families can replace some genes (e.g., *pdxR* can replace *pdxB* [20]). Some genes have paralogs with different functions, such as the *pdxA* paralog *pdxA2* [36, 37]. Ultimately the presence of *pdxS* and *pdxT* in each genome was the criterium used to predict the presence of an active DXP-I pathway. In contrast, the presence of *pdxJ* was used to predict the presence of a DXP-D pathway. If identifying signature genes for the *de novo* PLP synthesis pathways was straightforward, this was not the case for PLP salvage. Indeed, one of the salvage genes, *pdxH,* also encodes the last step of the *de novo* DXP-D pathway. The other salvage enzymes, such as the kinases (PdxK and PdxY), the phosphatase (YbhA), and the reductase PdxI, are all members of large superfamilies that are very difficult to annotate accurately. We did find that a majority (~86 %) of the genomes that encode PdxJ also harbour PdxH with or without PdxK/Y being present in the genome. Only ~9 % of the genomes analysed harbour only PdxH, and around 5 % harbour PdxH with PdxK or/and PdxY. This information suggests that PdxH belongs to the DXP-D synthesis pathway, not the salvage pathway per se. So, we decided to only use the presence of a PdxK/Y protein as a signature for the presence of the B_6_ salvage pathway in a given genome. Similar signature enzymes for DXP-D, DXP-I and salvage pathways were identified in the Rodionov study focusing on human gut bacteria [41].

**Fig. 4.**
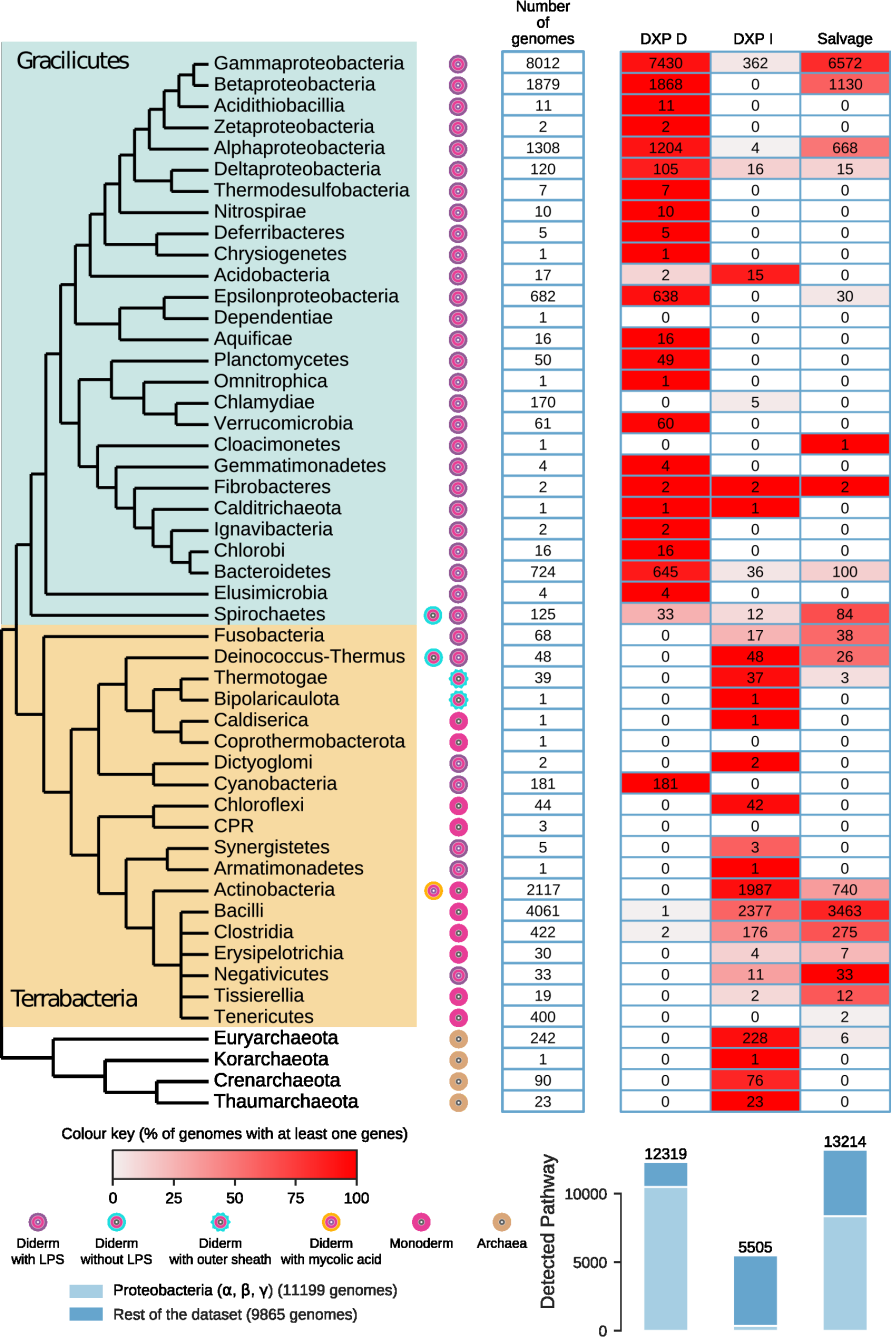
Taxonomic distribution of the PLP pathways in Bacteria and Archaea. Cells indicate the number of genomes with at least one detected pathway. The cell’s colour gradient represents the proportion of genomes with at least one pathway in the clade. The bar plot shows the total number of detected pathways. The bars are separated into alpha-, beta-, and gamma-proteobacteria versus the other clades. The cladogram symbolizes approximated relationships between the bacterial and archaeal taxa analysed in this study and was drawn using the phylogenetic analysis performed by Taib *et al*.[89].

Using these criteria, we predicted 12 319 DXP-D, 5505 DXP-I pathways, and 13 214 salvage pathways within 21 105 genomes (Fig. 4, Table S6A). Archaea only harbour the DXP-I pathway. On the bacterial side, ten bacterial genomes appeared to harbour both DXP-D and DXP-I pathways (* Acidiphilium cryptum* [alpha-proteobacteria], *Acidiphilium multivorum* [alpha-proteobacteria], *Caldithrix abyssi* [Calditrichaeota], *Desulfosarcina ovata* [delta-proteobacteria]*, Fibrobacter succinogenes* [Fibrobateres], *Methylophaga nitratireducenticrescens* [gamma-proteobacteria], *Minicystis rosea* [delta-proteobacteria], *Syntrophus aciditrophicus* [delta-proteobacteria], *Thiohalobacter* [gamma-proteobacteria], *Thiohalobacter thiocyanaticus* [gamma-proteobacteria]). Closer analysis of the genomes of these bacteria suggests the DXP-D pathway might not be complete. Indeed, they all were missing Epd proteins, and six were also missing PdxB/PdxR. In the case of two alpha-proteobacteria (* Acidiphilium cryptum *, *Acidiphilium multivorum *), the DXP-I pathway genes (*pdxS* and *pdxT*) are located on a plasmid suggesting a recent acquisition.

As PdxJ was used as a signature protein for the DXP-D pathway, genomes that lacked *pdxJ* genes were predicted to lack this pathway. However, we identified nine genomes lacking *pdxJ* but that harboured four of the six other DXP-D pathway genes. Further analysis revealed that the *pdxJ* genes had been missed because of contig assembly issues (see supplemental analyses section). These few cases support our choice of PdxJ as a signature protein for the presence of the DXP-D PLP pathway.

With a few exceptions discussed below, the distribution of one or the other PLP pathway broadly follows taxonomy with a split into two groups (Fig. 4). The DXP-D pathway is present almost exclusively in Gracilicutes (Gram-negative organisms). The only exception is Cyanobacteria, the only members of the Terrabacteria group that depend on the DXP-D pathway. The DXP-I is the predominant pathway present in Terrabacteria and all Archaea. Still, it is also found in Gracilicutes such as Acidobacteria and a small percentage of gamma or delta-proteobacteria (469 genomes). It seems that the observed taxonomic grouping of the DXP-D and DXP-I pathways follows the structural characteristics of the envelope. Species with two membranes (diderm) prefer the DXP-D pathway, while species with only one membrane (monoderm) prefer the DXP-I pathway.

A substantial number of organisms analysed (~10 %) like Chlamydiae, Tissierellia, Erysipelotrichia, and Tenericutes lacked the DXP-D and DXP-I pathways. However, many of these are predicted to harbour B_6_ salvage pathways, with ~1900 organisms harbouring only a PLP salvage pathway identified (Table S7). Furthermore, most of the corresponding genomes (~93 %) only encode a PdxK or PdxY kinase suggesting that *pdxH* is not used for these species and that they can salvage only PL. In contrast, the remaining organisms that harbour both PdxH and PdxK should be able to salvage the three non-phosphorylated B_6_ vitamers, PN, PL, and PM. However, as an uncharacterized B_6_ kinase could be present, the B_6_ vitamer requirements for the growth of given organisms should always be experimentally validated.

No PLP synthesis or salvage pathways could be predicted for 848 genomes corresponding to 269 unique NCBI taxonomic ids. A small subset of these encoded only a PdxT homolog (32 cases) or only a PdxS homolog (eight cases). Therefore, one cannot eliminate the possibility that in these species, only one of the two proteins is required to synthesize PLP, as recently shown in *B. subtilis* [69]. For the remaining genomes (Table S6C), it is likely that they encode a PLP transporter. PLP-dependent sulphur transferase IscS/SufS is the only PLP-dependent enzyme found in the minimal synthetic genome *Mycoplasma* JCV_Syn3 [12] suggesting it is one of the universally conserved PLP-dependent enzymes. Indeed, we detected IscS/SufS homologs in all organisms lacking PLP synthesis and salvage pathways (Table S8), confirming these must transport PLP by unknown mechanisms as no homolog of the only known PLP transporter, P5PA, was identified (data not shown).

### Many PLP *de novo* pathway genes are still to be discovered

We do not think that the analysis performed here is reliable enough to identify missing genes for the salvage pathways. As discussed above, alternative B_6_ kinase enzymes remain to be found. For the *de novo* synthesis pathways, most of the missing proteins are at the beginning of the DXP-D pathway, especially to replace Epd, SerC*,* or PdxB. For example, all Cyanobacteria encode DXS, PdxA, and PdxJ homologs (Fig. 3 and Table S5) but lack PdxB or PdxR and Epd. These pathway holes had already been identified in Cyanobacteria and the *Helicobacter */* Campylobacter* clades [70, 71]. Still, it is clear from our analysis that these genes are missing in most Gracilicutes clades, except the best-studied ones such as gamma-, beta- and alpha-proteobacteria. Indeed, organisms missing PdxB/PdxR are verified prototrophs such as *Desulfovibrio vulgaris* str. Hildenborough [72] or *Xanthomonas campestris* [73] must make PLP *de novo*. SerC/PdxF is similarly missing in many Gracilicutes. However, transaminases are notoriously promiscuous, and every genome usually encodes several homologs [74]. Hence, the PdxF orthologs are difficult to predict using only computational methods. Only experimental testing of substrate specificity of aminotransferase candidates [74] or TnSeq profiling data [75] will help resolve these missing gene cases.

Much more work is clearly required to understand the early part of the DXP-D pathway in Gracilicutes. Several scenarios can be envisioned to fill the identified pathway gaps. The existence of promiscuous enzymes that could partially replace portions of the canonical DXP-D pathway cannot be eliminated. Indeed, it has been reported that the DXP-D pathway can be implemented in a *B. subtilis pdxST* deletion mutant by expressing the *pdxJ* and *pdxH* genes from *E. coli *. Only two genomic alterations enable the bacteria to use the heterologous enzymes together with yet unknown promiscuous enzymes [76]. This might also be the case in bacteria/archaea that do not possess Epd and PdxR/PdxB orthologs. In addition, studies from the Copley laboratory have identified single mutations in different genes that could bypass the canonical pathways [77, 78] rediscovering the pioneer work from the Dempsey laboratory that pathways that use the 4-hydroxythreonine (4-HT) intermediate are viable solutions for *E. coli* mutants that lack *pdxB* (for review, see [79]). Such 4-HT-dependent pathways are yet to be found as the primary PLP synthesis route in natural settings [37] but can easily be engineered as shown in *B. subtilis* [80, 81].

Other possibilities rely on non-orthologous displacement, as seen in *R. meliloti* where the FAD-dependent oxidase PdxR (SMc00985) replaces the NAD-dependent PdxB. Analysis of TnSeq data available for *Desulfovibrio vulgaris* str. Hildenborough shows that the two genes that have the highest co-fitness scores with *pdxA* (*DVU2241*) and *pdxJ* (*DVU1908*) in conditions of B_6_ limitation are *DVU0826* and *DVU0827* (Fig. S5), annotated as encoding the two subunits of the glycolate oxidase complex, the iron-sulphur subunit (GlcF), and the FAD-dependent subunits respectively (GlcD). This co-fitness is conserved in another *D. vulgaris* strain (Fig. S5). DVU0827 is 33 % identical in sequence to *S. meliloti* PdxR (SMc00985). The proposal that DVU0826/DVU0827 replaces PdxB has independently been made by Trotter *et al*. [82].

**Fig. 5.**
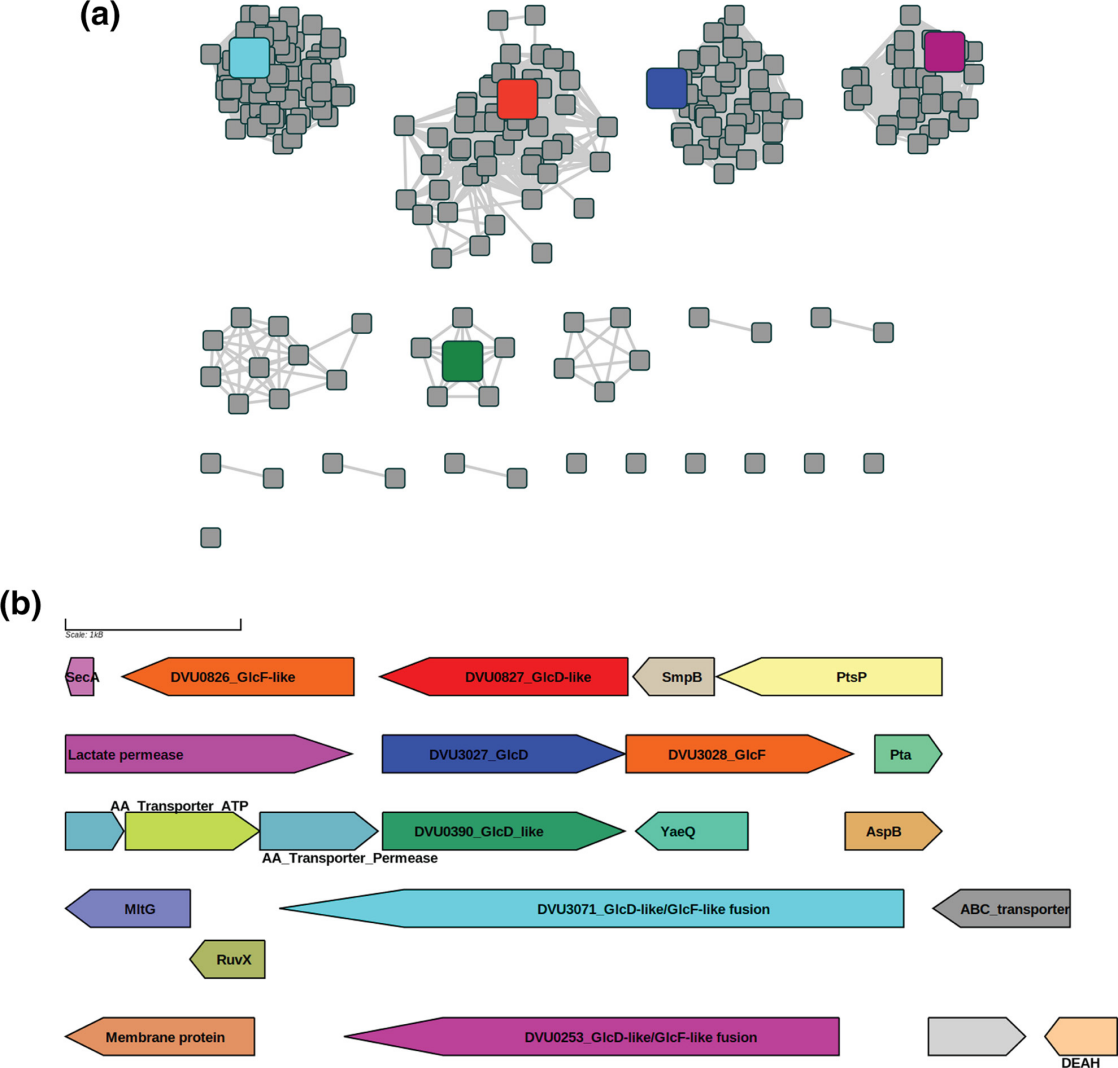
Paralogous groups of PF02913, FAD-linked oxidase (glycolate oxidase superfamily). Panel (a). Sequence similarity network (SSN) of members of PF02913 in Desulfovibrionales displayed with an alignment score of 140 (nodes are UniProt IDs) that separates the paralogous groups. Red node, DVU0827 (UniProt ID Q72DV2); blue node, DVU3027 (UniProt ID Q726S9); green node, DVU0390 (UniProt ID Q72F25); magenta node, DVU0253 (UniProt ID Q72FG1); cyan node, DVU03071 (UniProt ID Q726N5). Panel (b). Genome context () for the paralogs in *Desulfovibrio vulgaris* str. Hildenborough (NC_002937.3), colour coding for five paralogs conserved between the two panels. AA_Transporter_ATP, Amino Acid ABC transporter ATPase component, AA_Transporter_permease: Amino Acid ABC transporter permease component; DEAH, DEAH helicase domain.

The glycolate oxidase family is complex with many paralogs and redundant systems [83]; hence it is impossible to propagate the proposed 4-phosphoerythronate dehydrogenase annotation for DVU0826/DVU0827 to the correct family subgroup without further analyses. We, therefore, performed a Sequence Similarity Network (SSN) [53] of the Pfam family (PF02913) corresponding to the FAD-dependent GlcD subunit. This analysis identified multiple paralogous groups in Desulfovibrionales (Fig. 5, panel A). *Desulfovibrio vulgaris* str. Hildenborough encodes five paralogs, DVU0253 (UniProt ID Q72FG1), DVU0390 (UniProt ID Q72F25), DVU0827 (UniProt ID Q72DV2), DVU3027 (UniProt ID Q72659), and DVU3071 (UniProt ID Q726N5). The genes encoding DVU0827 and DVU3027 are each in an operon with a gene encoding their iron-sulphur subunits, DVU0826, and DVU3028, respectively (Fig. 5, panel B). DVU0390 is in an operon with genes encoding a putative amino acid transporter. DVU0253 and DVU0371 are multidomain proteins with additional FAD-binding and iron-sulphur domains. The only subgroup with relevant neighbourhood information revealed by the Gene Neighbourhood Network (GNN), was the linking of members of the DVU0827 subgroup with lactate catabolism enzymes (Fig. 5b and data not shown). Previous genetic studies have indeed shown that DVU3027/3028 are involved in d-lactate catabolism but not l-lactate utilization [83]. As expected in light of the newly predicted functional role in B_6_ synthesis, deleting the DVU0826/0827 genes does not affect growth on d-lactate nor l-lactate, a result confirmed by the TnSeq data (Fig. S5). We, therefore, suggest propagating the pdxR/4-phosphoerythronate dehydrogenase annotation to the DVU0827 subgroup, while tagging the d-lactate oxidase role to the DVU3027 subgroup (Table S9).

The SSN analysis of the PF02913 family also revealed that this annotation could not be propagated to other subgroups without additional experiments. For example, the *R. meliloti* PdxR (UniProt ID F7XD40) is in another cluster in the SSN generated for Bacteria superkingdom (Fig. S6). In addition, analysis of d/l-lactate catabolism in *Geobacter sulfurreducens* showed that the glycolate oxidase-like subunits GSU3296/GSU3297 (UniProt ID Q747H0) are not involved in lactate metabolism and must have a housekeeping function [84]. As *pdxB* is missing in this organism (Table S5), it is tempting to propose that GSU3296/97 fulfils this function. However, GSU3296 is in the same sequence cluster as a SwissProt-curated glycolate oxidase (Fig. S6); hence it is impossible to annotate without further experimental validation steps. In summary, if we can fill the *pdxB* pathway hole in Desulfovibrionales, the gene is still missing in many Gracilicutes clades and nearly all cyanobacteria.

The second part of the DXP-D pathway is less problematic. Indeed, *pdxJ* is well annotated and was used as the pathway signature gene. The distribution analysis suggests that PdxA2 might be used as a PLP synthesis gene when PdxA is missing, as we have previously shown that PdxA2 does catalyse the PdxA reaction leaving very few holes paralogs [37]. However, the last enzyme of the DXP-D pathway, PdxH, is missing in nearly 1000 genomes that encode both PdxA and PdxJ (Table S5). Over half of the genomes are in the *Campylobacter/Helicobacter* clades. Analysis of the literature showed that a metabolic reconstruction had annotated the CJJ81176_0462 gene as PdxH in *Campylobacter jejuni* subsp. jejuni 81–176 clades [71, 72]. This gene encodes a member of the COG5015 family annotated as ‘Uncharacterized protein, pyridoxamine 5'-phosphate oxidase (PNPOx-like) family’. However, as noted in the Pfam description of this family (PF01243), the function has not been validated. The only members of fungal origin that were experimentally tested lacked PdxH activity [85]. Another complication is the discovery of a PdxH paralog, PhzG, involved in phenazine synthesis [86]. We were therefore conservative until more bacterial members of the COG5015 family were tested and did not include this family in our PLP synthesis set.

### The distribution of PLP synthesis genes on plasmids reveals the plasticity of PLP metabolism

The genomes of *Azospirillum brasilense* and *Azospirillum sp*. TSH58 were of specific interest as four of the DXP-D pathway genes (*dxs*, *pdxA*, *pdxJ*, and *pdxR*) were encoded on plasmids while *serC* was encoded on the main chromosome, suggesting these two genomes had acquired the DXP-D pathway genes by a horizontal transfer through this plasmid. Similarly, the *Azospirillum* sp. CFH genome encodes *serC*, *pdxA*, and *pdxJ* on its plasmid and *dxs* and *pdxR* on its chromosome. This observed gene organization suggests that genes encoding for the PLP synthesis pathways, usually scattered around the chromosome, can be transferred on a cluster in a transferable plasmid. The *pdxS* and *pdxT* gene pair were also found in plasmids of *Acidiphilium cryptum* JF-5 and *Acidiphilium multivorum* AIU301 (alpha-proteobacteria), *Eubacterium eligens* ATCC 27750 (Clostridia), *Halanaeroarchaeum sulfurireducens* (Euryarchaeota), *Pasteurella multocida* subsp. Multocida (gamma-proteobacteria), this suggesting a possible mechanism by which this minimal DXP-I pathway could be transferred to new organisms.

## Conclusions

This comprehensive analysis of PLP synthesis salvage genes in Bacteria and Archaea confirmed previous studies showing the high number of non-orthologous displacements for enzymes of core metabolism, even for pathways that are thought to be very conserved like those of vitamins [35]. This makes the prediction of pathways using automatic metabolic reconstruction tools both problematic and useful to identify pathway holes that need to be filled. The process of identifying these missing genes is still laborious but the combination computational tools with TnSeq experiments as performed for *D. vulgaris* (this work and [83]) can greatly accelerate these discoveries.

Another interesting observation was the number of genomes (>850) lacking all synthesis and salvage genes (Table S6C). The corresponding organisms, which are mainly intracellular bacteria like *Mycoplasma *, *Rickettsia* or *Chlamydiae *, must hence transport the final PLP molecule unless an unknown kinase allows the salvage of B_6_ vitamers. This knowledge could help in the design of culture media but also shows how antibacterial agents that would target the PLP synthesis enzyme could be ineffective against certain pathogens. This type of derived information would also be valid for all the predicted B_6_ auxotrophs that rely on known salvage enzymes (Tables S6B and S7).

Finally, in this study we did not expand on the previous analysis performed by the Rodionov group on transporters and regulators involved in PLP metabolism [28], as the annotations of the few known transporters are difficult to propagate because they are members of large superfamilies. Another possibility is that as PL is highly reactive [87], the vitamer could react extracellularly with amino acids/peptides that are taken up by microbes together with the attached B_6_ vitamer. This could explain why a specific B_6_ transporter has never been identified. Another possibility is that B_6_ is transported as a sugar adduct as these are abundant in nature [88]. A combination of experimental and computational studies will be required to solve the B_6_ transporter puzzles in most sequenced prokaryotes.

## Supplementary Data

Supplementary material 1

Supplementary material 2

Supplementary material 3

Supplementary material 4

Supplementary material 5

Supplementary material 6

Supplementary material 7

Supplementary material 8

Supplementary material 9

Supplementary material 10
