# Supplementary material for: Pyridoxal 5’-phosphate synthesis and salvage in Bacteria and Archaea: predicting pathway variant distributions and holes
Source: Microb Genom. 2023 Feb 2;9(2):mgen000926. doi: 10.1099/mgen.0.000926 (PMC9997740; doi:10.1099/mgen.0.000926)

Pyridoxal 5'-phosphate synthesis and salvage in Bacteria and Archaea: predicting pathway variant distributions and holes

Rémi Denise[1], Jill Babor[1], John A. Gerlt [2] and Valérie de Crécy-Lagard[1,3]*

[1]Department of Microbiology and Cell Sciences,

[2]Department of Chemistry

[3]Genetics Institute, University of Florida, Gainesville, FL 32611, USA

*Correspondence: vcrecy@ufl.edu, V dC-L.

Supplemental Methods

The full pipeline summarized in Figure 2 is described below. We proceeded in two steps to generate the final HMM profiles used in the proteome analysis. First, we did an exploratory search using the HMM profiles already developed in the Panther v15 databases [1] for all PLP pathway proteins and the superfamily, using hmmsearch (default parameters) from HMMER package v3.3 [2], (Table S3). Only the hits with an e-value superior to 1e-6 and a 50% coverage of the smallest between the profile or the sequence were conserved. In cases where multiple profiles hit the same sequence after the first threshold, we chose the profile with the best e-value to annotate the sequence.

All the detected proteins for each protein of the PLP pathways were compiled and the number of sequences was reduced below 1500 for each protein family based on the genus rank and the number of copies in the genomes (*i.e.*, the genome for the same genus with a higher number of copies). Using the reduced sets, the tree for each protein was inferred as described in the Method section. Then the monophyletic groups were identified in the tree using annotations from KofamKOALA [3,4] and eggNOG-Mapper v2 [5] as guides and extracted the sequences in different files for each group. Using these annotations and the phylogenetic placement of the sequence in the tree, we were able to identify monophyletic groups with the same functional annotation. The example of the Ribokinase superfamily tree (Fig. S3) shows how we can separate the sequences

annotated as PdxK, PdxY, RbsK, ThiD, and ThiD2, a subfamily used in bacilli as a PdxK replacement (named pdxK_basu in our analysis). Once the subfamilies had been defined, these were used as seeds to create new HMM profiles. The different seed sequences were aligned using MAFFT v7.453 [6] (linsi algorithm, using the group's subtree as guide tree, option --treein). The poorly aligned regions at the extremities of the alignment were manually trimmed in the alignment using SEAVIEW v5.0.2 [2]. The trimmed alignments were used to build the HMM profile using hmmbuild (default parameters) from HMMER package v3.3 [2], resulting in a set of 113 HMM protein profiles (Table S4).

In the second round of refinements of the HMM profiles, we ran hmmsearch (default parameters) from HMMER package v3.3 [2] using the 113 HMM protein profiles against the same database and we decided to keep all the hits with an e-value > 1e-6 and a coverage of the alignment >= 50%. Using these sequences, phylogenetical trees were inferred for each annotated protein group as described in the Methods section. In parallel, each set of sequences was divided in groups using the EFI-EST sequence similarity network [7] using an alignment score threshold corresponding to 35 to 40% pairwise identity. Each tree was then divided into sub-trees using the clusters identified by the SSNs and rarefied to reduce their size and redundancy to 20 representatives by group using Treemmer v0.3 [8] (option -X 20). Each protein sequence of the representatives belonging to the same superfamily was concatenated into the same multi-fasta file. The superfamily tree was then inferred as described in the method section. For each superfamily, groups inside the superfamily were separated using EFI-EST SSN with a step-by-step evolution of the alignment score threshold to identify the best threshold for each superfamily to separate PLP pathway protein from the other functional groups (supplemental data 1 in FigShare [9]). After mapping the SSN groups on the tree of each superfamily to make sure that the groups were homogenous (supplemental data 2 in FigShare [9])._ HMM protein profiles were built for each group as described below.

HMM protein profile generation

An initial alignment was performed using MAFFT v7.453 [10] ("linsi" algorithm, using the tree of the superfamily as guide tree, option --treein). Then, the resulting multiple alignments were analyzed using BMGE v1.12 [11] (options -m BLOSUM62-g 0.2 and -oh

to have an HTML output). Next, the HTML output was parsed to identify the non-conserved block at the border of the alignment and trimed them out. Finally, the trimmed alignment was used to build the HMM profile using hmmbuild (default parameters) from HMMER package v3.3 [2]. All final profiles are given in Supplemental data 3 in FigShare [9].

Sequence similarity networks (SSNs) and genome context

SSNs for PF02913 (UniProt Release 2022_01) were generated using the EFI-EST web tool [7]. For the Desulfovibrionales Order (Fig. 5, panel A), the SSN was generated using UniProt IDs; an alignment score of 140 that separates paralogous groups was used to display the SSN. For the Bacteria Superkingdom (Fig. 5, panel C), the SSN was generated using UniRef90 clusters; an alignment score of 140 was used to display the SSN. Genome neighborhood diagrams (GNDs) were generated for the five paralogs in *Desulfovibrio vulgaris* str. Hildenborough using the EFI-GNT web tool [7].

Supplemental Analyses

Analyses of the missing PdxJ proteins

Eight genomes seemed to harbor most of the DXP-dependent pathway genes except *pdxJ*; the corresponding species were *Brucella ceti TE28753-12*, *Escherichia coli* (NZ_LR134247), *Pseudomonas aeruginosa VRFPA04*, *Pseudomonas aeruginosa* (NZ_CP022002), *Pseudomonas sp. AK6U*, *Ralstonia solanacearum* (NZ_CP023016), *Salmonella enterica subsp. enterica serovar Waycross*, *Sphingomonas sp. JJ-A5*, *Tannerella forsythia 3313*. All these genomes harbored *pdxA, pdxB/R*, *dxs,* and *serC*. and five of those also harbored *epd*, suggesting the *pdxJ* gene might have been missed. Using tblastn (v2.11.0+, default parameters) [12,13], we could detect, in 4 of the genomes, *pdxJ* in two fragments suggesting an artifactual frameshift was causing the absence of this protein in the final Refseq file. Furthermore, for one of the genomes, *Pseudomonas aeruginosa VRFPA04,* we saw that the genome version in contig in PATRIC harbors the missing *pdxJ* in the gene set. We could not find any trace of *pdxJ* in the three remaining genomes but anticipate this must be caused by problems in genome assembly as the same species/genus harbor *pdxJ* in their genomes.

References

1 Matsui, M. and Iwasaki, W. (2020) Graph splitting: a graph-based approach for superfamily-scale phylogenetic tree reconstruction. *Syst. Biol* 69, 265–279

2 Eddy, S.R. (2011) Accelerated profile HMM searches. *PLoS Computational Biology* 7, 1002195

3 Huerta-Cepas, J. *et al.* (2017) Fast genome-wide functional annotation through orthology assignment by eggnog-mapper. *Mol Biol Evol* 34, 2115-2122.

4 Huerta-Cepas, J. *et al.* (2018) EggNOG 5.0: a hierarchical, functionally and phylogenetically annotated orthology resource based on 5090 organisms and 2502 viruses. *Nucleic Acids Res* 47, 309–314

5 Katoh, K. and Standley, D.M. MAFFT multiple sequence alignment software version 7: improvements in performance and usability. *Mol Biol Evol* 30, 772-80

6 Gouy, M. *et al.* (2010) SeaView Version 4: A multiplatform graphical user interface for sequence alignment and phylogenetic tree building. *Mol. Biol. Evol* 27, 221–224

7 Zallot, R. *et al.* (2019) The EFI web resource for genomic enzymology tools: leveraging protein, genome, and metagenome databases to discover novel enzymes and metabolic pathways. *Biochemistry* 58, 4169–4182

8 Menardo, F. *et al.* (2018) Treemmer: A tool to reduce large phylogenetic datasets with minimal loss of diversity. *BMC Bioinformatics* 19, 164

9 https://microbiology.figshare.com/submit

10 Dress, A.W.M. *et al.* (2008) Noisy: Identification of problematic columns in multiple sequence alignments. *Algorithms Mol Biol* 3, 7

11 Criscuolo, A. and Gribaldo, S. (2010) BMGE (Block Mapping and Gathering with Entropy): a new software for selection of phylogenetic informative regions from multiple sequence alignments. *BMC Evol Biol* 10, 210

12 Camacho, C. *et al.* (2009) BLAST+: Architecture and applications. *BMC Bioinformatics* 10,

13 Altschul, S.F. *et al.* (1990) Basic local alignment search tool. *Jl Mol Biol*215, 403–410

Supplemental Figures

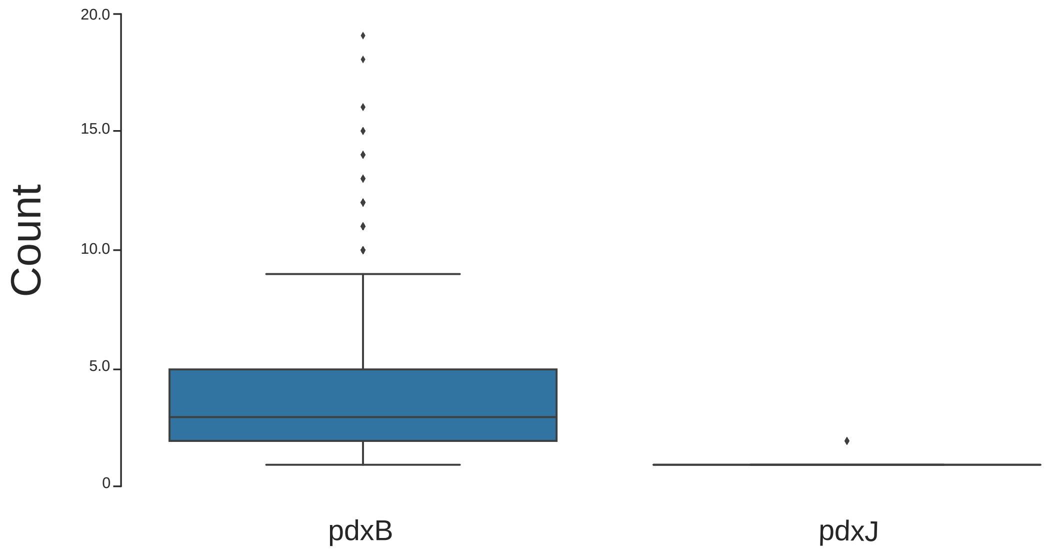

Figure S1. Difference between the number of hits of the hmm profile for PdxB and PdxJ proteins. This box plot shows the difference between the number of proteins detected in the same genome for two genes belonging to the PLP pathway (*pdxJ* and *pdxB*). This shows a disparity between the number of homologs of some proteins in the genomes and the importance of trying to annotate all the homologs to correctly annotated the protein of interest.

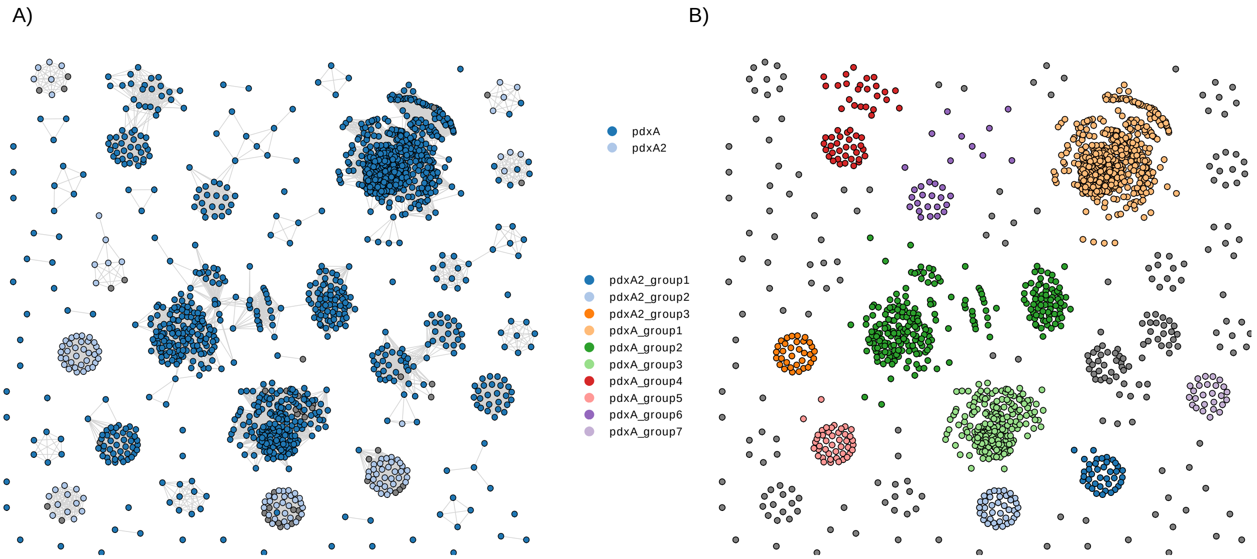

Figure S2: Sequence similarity network (SSN) representation at the alignment score threshold that allows the separation of PdxA and PdxA2 subfamilies. Each node represents a protein, and each edge the alignment score from the blast all vs. all comparison. A) SSN annotates using the KOFAM HMM profile to annotate the proteins. The grey nodes are proteins that were not annotated as PdxA or PdxA2. B) Same SSN with the selected cluster in color. Only the cluster with more than 20 non-identical sequences was used to create HMM profiles.

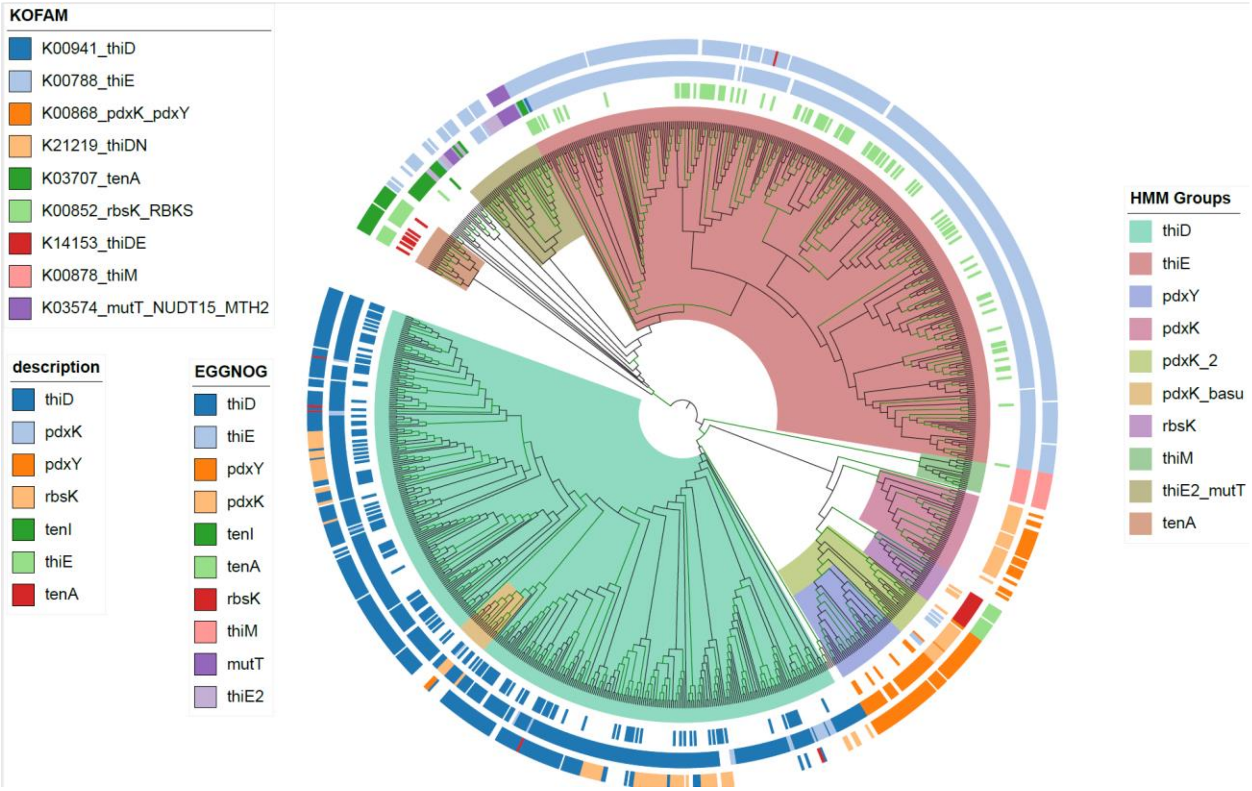

Figure S3. Mid-rooted phylogeny of the Ribokinase superfamily. The tree was built with all the protein sequences detected using the PdxH, PdxK, PdxY, ThiD HMM profiles. The branches are in green if the ultrafast bootstrap is >95%. The different colored stripes indicate the annotation of the proteins with NCBI description, EGGNOG, and KOFAM. The highlight color of the branch represents the selected group of sequences used to create the new HMM profile. The tree was built using IQ-Tree, 10,000 replicates of UFBoot.

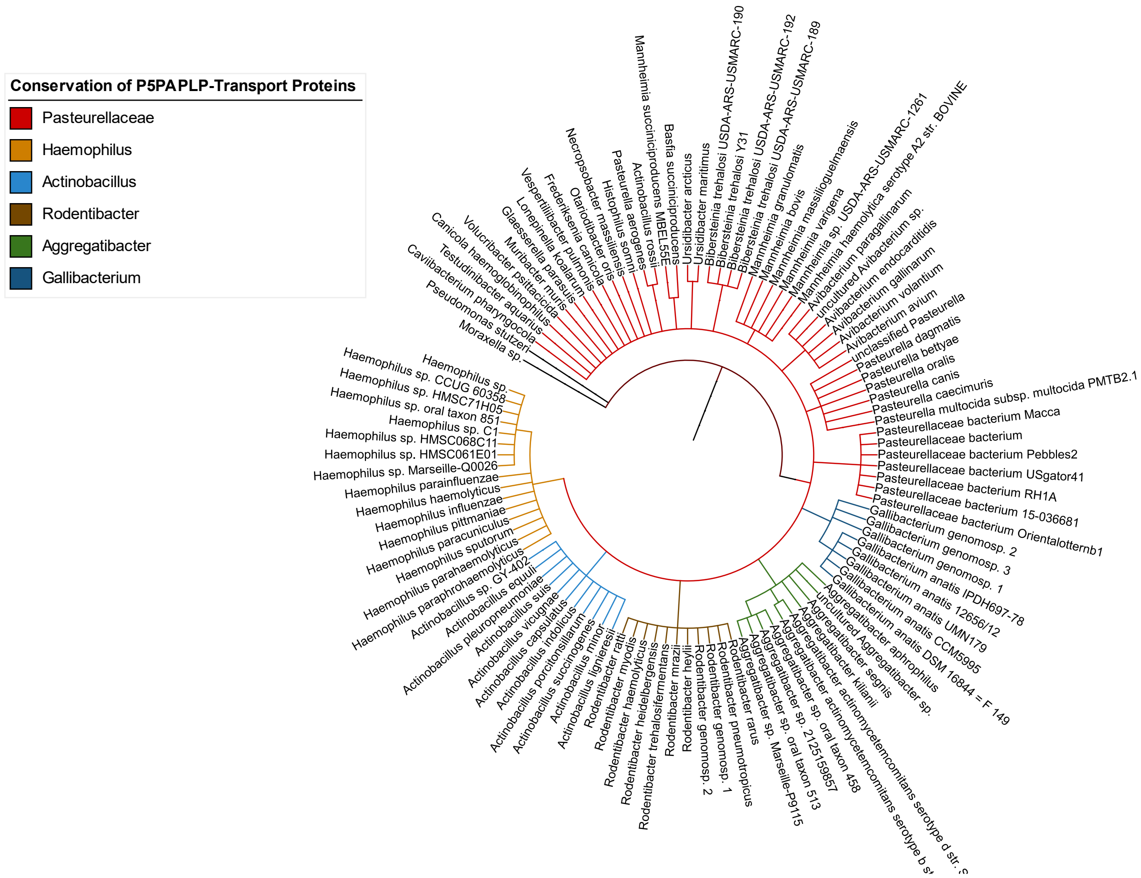

Figure S4. Conservation of P5PA PLP-transporting protein. Blastp was used to find homologs of the *Acinobacillus pleuropneumoniae* P5PA transporter protein, using four iterations of Psi-Blast and WP_005596767.1 as the input query. P5PA is conserved only in a select group of gamma-proteobacteria, most belonging to pathogenic *Pasteurellaceae* (red branch). Several species of a particular genus were found to all have P5PA homologs and their clade branches are colored accordingly: Haemophilus (mustard), Actinobacillus (light blue), Rodentibacter (brown), Aggregatibacter (green), Gallibacterium (dark blue).

Figure S5. TnSeq data from the Fitness Browser (https://fit.genomics.lbl.gov/cgi-bin/myFrontPage.cgi) showing the importance of the DVU0827 and DVU0826 for growth in the absence of Vitamin B_6. The specific data is available at the following links: https://fit.genomics.lbl.gov/cgi-bin/cofit.cgi?orgId=DvH&locusId=207725, https://fit.genomics.lbl.gov/cgi-bin/cofit.cgi?orgId=DvH&locusId=207377 and https://fit.genomics.lbl.gov/cgi-bin/cofit.cgi?orgId=Miya&locusId=8499511

Experiment set12IT079 for Desulfovibrio vulgaris Hildenborough JW710

MoLS4 without Pyridoxine Hydrochloride

Genes with specific phenotypes:

gene	name	fitness	t score	description	
DVU2241	pdxA	-1.6	-8.8	pyridoxal phosphate biosynthetic protein PdxA (TIGR)	compare
DVU0827		-1.4	-6.7	glycolate oxidase, subunit GlcD, putative (TIGR)	compare
DVU0826		-1.3	-6.6	glycolate oxidase, iron-sulfur subunit, putative (TIGR)	compare

Top cofit genes for DVU1908 from Desulfovibrio vulgaris Hildenborough JW710

pdxJ: pyridoxal phosphate biosynthetic protein PdxJ (TIGR)
SEED: Pyridoxine 5'-phosphate synthase (EC 2.6.99.2)
KEGG: pyridoxine 5-phosphate synthase

Rank	Hit	Name	Description	Conserved?	Cofitness
1	DVU0826		glycolate oxidase, iron-sulfur subunit, putative (TIGR)	0.78	0.37
2	DVU2241	pdxA	pyridoxal phosphate biosynthetic protein PdxA (TIGR)	no	0.36
3	DVU0827		glycolate oxidase, subunit GlcD, putative (TIGR)	0.87	0.35

Top cofit genes for DvMF_0281 from Desulfovibrio vulgaris Miyazaki F

pyridoxine 5'-phosphate synthase (RefSeq)
SEED: Pyridoxine 5'-phosphate synthase (EC 2.6.99.2)
KEGG: pyridoxine 5-phosphate synthase

Rank	Hit	Name	Description	Conserved?	Cofitness
1	DvMF_2875		FAD linked oxidase domain protein (RefSeq)	0.35	0.87
2	DvMF_2874		4Fe-4S ferredoxin iron-sulfur binding domain protein (RefSeq)	0.37	0.78

Figure S6. SSN for PF02913 for superkingdom Bacteria displayed with an alignment score of 140 (nodes are UniRef90 clusters). Red node, DVU0827; blue node, *R. meliloti* PdxR (UniProt F7XD40); green node GSU_3296 (Q747H0).

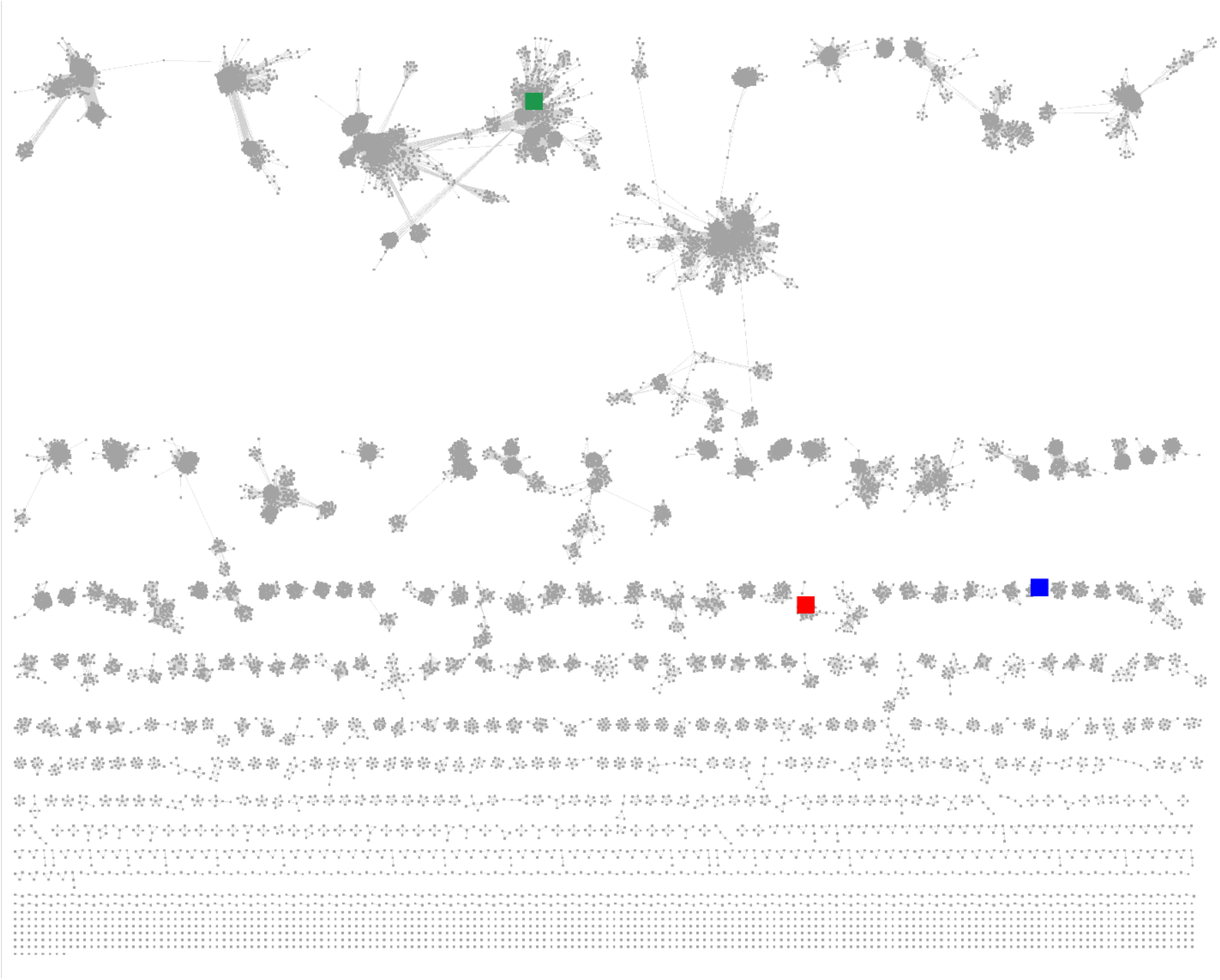

Supplement: Supplementary material 1 [file mgen-9-926-s001.pdf]
